# Maternal tobacco smoking and offspring autism spectrum disorder or traits in ECHO cohorts

**DOI:** 10.1002/aur.2665

**Published:** 2022-02-24

**Authors:** Irva Hertz‐Picciotto, Susan A. Korrick, Christine Ladd‐Acosta, Margaret R. Karagas, Kristen Lyall, Rebecca J. Schmidt, Anne L. Dunlop, Lisa A. Croen, Dana Dabelea, Julie L. Daniels, Cristiane S. Duarte, M. Daniele Fallin, Catherine J. Karr, Barry Lester, Leslie D. Leve, Yijun Li, Monica McGrath, Xuejuan Ning, Emily Oken, Sharon K. Sagiv, Sheela Sathyanaraya, Frances Tylavsky, Heather E. Volk, Lauren S. Wakschlag, Mingyu Zhang, T. Michael O'Shea, Rashelle J. Musci, program collaborators for Environmental influences on Child Health Outcomes (ECHO)

**Affiliations:** ^1^ Department of Public Health Sciences and MIND Institute University of California, Davis School of Medicine Davis California USA; ^2^ Channing Division of Network Medicine, Brigham and Women's Hospital Harvard Medical School Boston Massachusetts USA; ^3^ Department of Epidemiology Johns Hopkins Bloomberg School of Public Health Baltimore Maryland USA; ^4^ Department of Epidemiology Geisel School of Medicine at Dartmouth Hannover New Hampshire USA; ^5^ A.J. Drexel Autism Institute Drexel University Philadelphia Pennsylvania USA; ^6^ Department of Gynecology & Obstetrics Emory University School of Medicine Atlanta Georgia USA; ^7^ Division of Research Kaiser Permanente Oakland California USA; ^8^ LEAD Center and Department of Epidemiology Colorado School of Public Health Aurora Colorado USA; ^9^ Departments of Epidemiology and Maternal and Child Health; Gillings School of Global Public Health University of North Carolina Chapel Hill North Carolina USA; ^10^ Department of Psychiatry Columbia University, New York State Psychiatric Institute New York New York USA; ^11^ Department of Mental Health Johns Hopkins Bloomberg School of Public Health Baltimore Maryland USA; ^12^ Departments of Pediatrics and Environmental & Occupational Health Sciences University of Washington Seattle Washington USA; ^13^ Brown Center for the Study of Children at Risk and Departments of Psychiatry and Human Behavior and Pediatrics, Alpert Medical School, Brown University Women and Infants Hospital in Rhode Island Providence Rhode Island USA; ^14^ College of Education University of Oregon Eugene Oregon USA; ^15^ Department of Population Medicine Harvard Medical School and Harvard Pilgrim Health Care Institute Boston Massachusetts USA; ^16^ Center for Environmental Research and Children's Health University of California, Berkeley, School of Public Health Berkeley California USA; ^17^ Department of Pediatrics, Seattle Children's Research Institute University of Washington Seattle Washington USA; ^18^ Department of Preventive Medicine University of Tennessee Health Science Center Memphis Tennessee USA; ^19^ Department of Medical Social Sciences, Feinberg School of Medicine, and Institute for Innovations in Developmental Sciences Northwestern University Chicago Illinois USA; ^20^ Department of Pediatrics University of North Carolina at Chapel Hill School of Medicine Chapel Hill North Carolina USA; ^21^ See Acknowledgments for full listing of collaborators

**Keywords:** autism spectrum disorder, children, maternal smoking, prenatal tobacco use

## Abstract

Given inconsistent evidence on preconception or prenatal tobacco use and offspring autism spectrum disorder (ASD), this study assessed associations of maternal smoking with ASD and ASD‐related traits. Among 72 cohorts in the Environmental Influences on Child Health Outcomes consortium, 11 had ASD diagnosis and prenatal tobaccosmoking (*n* = 8648). and 7 had Social Responsiveness Scale (SRS) scores of ASD traits (*n* = 2399). Cohorts had diagnoses alone (6), traits alone (2), or both (5). Diagnoses drew from parent/caregiver report, review of records, or standardized instruments. Regression models estimated smoking‐related odds ratios (ORs) for diagnoses and standardized mean differences for SRS scores. Cohort‐specific ORs were meta‐analyzed. Overall, maternal smoking was unassociated with child ASD (adjusted OR, 1.08; 95% confidence interval [CI], 0.72–1.61). However, heterogeneity across studies was strong: preterm cohorts showed reduced ASD risk for exposed children. After excluding preterm cohorts (biased by restrictions on causal intermediate and exposure opportunity) and small cohorts (very few ASD cases in either smoking category), the adjusted OR for ASD from maternal smoking was 1.44 (95% CI, 1.02–2.03). Children of smoking (versus non‐smoking) mothers had more ASD traits (SRS T‐score + 2.37 points, 95% CI, 0.73–4.01 points), with results homogeneous across cohorts. Maternal preconception/prenatal smoking was consistently associated with quantitative ASD traits and modestly associated with ASD diagnosis among sufficiently powered United States cohorts of non‐preterm children. Limitations resulting from self‐reported smoking and unmeasured confounders preclude definitive conclusions. Nevertheless, counseling on potential and known risks to the child from maternal smoking is warranted for pregnant women and pregnancy planners.

**Lay Summary:**

Evidence on the association between maternal prenatal smoking and the child's risk for autism spectrum disorder has been conflicting, with some studies reporting harmful effects, and others finding reduced risks. Our analysis of children in the ECHO consortium found that maternal prenatal tobacco smoking is consistently associated with an increase in autism‐related symptoms in the general population and modestly associated with elevated risk for a diagnosis of autism spectrum disorder when looking at a combined analysis from multiple studies that each included both pre‐ and full‐term births. However, this study is not proof of a causal connection. Future studies to clarify the role of smoking in autism‐like behaviors or autism diagnoses should collect more reliable data on smoking and measure other exposures or lifestyle factors that might have confounded our results.

## INTRODUCTION

Autism spectrum disorder (ASD) is a developmental disorder, often highly disabling, that affects an estimated 1 in 44 children in the United States (U.S.) (Maenner et al., 2021). The observed prevalence of ASD has increased over the past 2 decades; hence, identifying modifiable risk factors is critical to addressing the public health impact of ASD. Epidemiological studies consistently have reported associations between air pollutants and an increased risk of ASD (Flores‐Pajot et al., 2016; Kalkbrenner et al., 2014). The constituents of air pollutants and tobacco smoke are similar although maternal smoking during pregnancy more directly exposes the fetus. It is surprising, then, that an association with ASD has not been reliably identified for maternal prenatal smoking, which has an estimated prevalence of 9–16% in the U.S. (Jamal et al., 2018).

Evidence on the association between maternal smoking and ASD has been conflicting, with some studies reporting harmful effects, and others, protective. Two meta‐analyses, reflecting the highly discordant results, estimated an essentially null odds ratio (OR) (1.02) and almost identical confidence intervals (CIs) (0.93–1.12 [Rosen et al., 2015] and 0.93–1.13 [Tang et al., 2015]). Sensitivity indicators suggested that these results were robust and free from publication bias.

Despite consistency of meta‐analyses, key methodologic limitations in the original studies bring their conclusions into question. Of the 15 studies considered by Tang et al. (2015), 6 did not adjust for confounders. Four adjusted for either birthweight or gestational age, two potential intermediates on a causal pathway linking maternal smoking with ASD (Abel et al., 2013; Abraham et al., 2017; Agrawal et al., 2018; Moore et al., 2019). Adjustment for either variable is likely to result in a biased estimate of the total effect of preconception/prenatal smoking on risk for ASD (Robins & Greenland, 1992). The present study from the National Institutes of Health‐funded Environmental Influences on Child Health Outcomes (ECHO) program sought to systematically address confounding in a standardized approach (Gillman & Blaisdell, 2018).

ECHO brings together U.S. cohorts of children with longitudinal follow‐up, harmonizes previously collected data, and introduces a common data collection protocol for continued follow‐up in a multisite collaborative research program. Here, we include cohorts with existing data on maternal smoking during pregnancy and outcomes of either an ASD diagnosis or the Social Responsiveness Scale (SRS), a measure of social impairments characteristic of the autism spectrum. Multiple large studies were amassed to carefully adjust for confounding, help reconcile apparent differences across studies of maternal smoking and ASD risk and illuminate potential reasons for discrepancies.

## METHODS

### Study population

Launched in 2016, the ECHO program investigates the influence of early life exposures on child health and development (Blackwell et al., 2018; Gillman & Blaisdell, 2018; Jacobson et al., 2018). Of the 72 extant cohorts in the ECHO program (https://www.nih.gov/echo/pediatric-cohorts), 13 included ASD diagnosis or traits and prenatal tobacco smoking, and 7 had SRS T‐scores. Five of these cohorts contributed to both the ASD and SRS analyses. For the analysis of ASD, two cohorts lacking key confounders or with 0 cases were excluded, leaving 11 cohorts for the pooled analysis of diagnoses. Exclusion of children missing either maternal prenatal smoking or key confounders resulted in 8648 (of 12,155) and 2399 (of 2682) children for the meta‐analyses of, respectively, ASD diagnosis and SRS T‐scores. Participating cohorts encompassed both general population studies that recruited pregnant women or recent deliveries and other designs that oversampled children with ASD, either via an initial case–control study or by restricting to pregnant women whose offspring were at a higher risk for ASD (e.g., preterm deliveries or children with first‐degree relatives who had ASD). Protocols for all cohorts were reviewed and approved by their local institutional review board.

### Variables

Two outcomes were examined—ASD diagnosis and SRS T‐scores. Some cohorts obtained both, whereas others obtained just ASD diagnosis (Table 1) or SRS T‐scores (Table 2). Separate analyses were conducted for the two different outcomes.

**TABLE 1:** Participant characteristics by cohorts included in the analysis of autism spectrum disorder (ASD) diagnosis

	Cohort 1	Cohort 2	Cohort 3	Cohort 4	Cohort 5	Cohort 6	Cohort 7	Cohort 8	Cohort 9	Cohort 10	Cohort 11
*n*	1177	142	2208	1900	1503	510	176	397	1366	885	1891
Type of study population	General population *n* (%)	General population *n* (%)	General population *n* (%)	ASD cases oversampled *n* (%)	General population *n* (%)	ASD enriched risk (preterm birth) *n* (%)	General population *n* (%)	ASD familial enriched risk *n* (%)	General population *n* (%)	ASD enriched risk (preterm birth) *n* (%)	General population *n* (%)
Child female	562 (47.7)	66 (46.5)	1080 (48.9)	416 (21.9)	726 (48.3)	239 (46.9)	87 (49.4)	151 (38.0)	670 (49.0)	434 (49.0)	882 (46.6)
Missing	0 (0.0)	0 (0.0)	0 (0.0)	0 (0.0)	41 (2.7)	0 (0.0)	0 (0.0)	26 (6.5)	0 (0.0)	0 (0.0)	0 (0.0)
Year of birth of child											
Before 1991	0 (0.0)	0 (0.0)	0 (0.0)	0 (0.0)	0 (0.0)	0 (0.0)	0 (0.0)	0 (0.0)	0 (0.0)	0 (0.0)	0 (0.0)
1991–2000	0 (0.0)	0 (0.0)	0 (0.0)	218 (11.5)	0 (0.0)	0 (0.0)	0 (0.0)	0 (0.0)	536 (39.2)	0 (0.0)	0 (0.0)
2001–2010	148 (12.6)	81 (57.0)	0 (0.0)	1470 (77.4)	1096 (72.9)	0 (0.0)	144 (81.8)	179 (45.1)	830 (60.8)	885 (100.0)	0 (0.0)
After 2010	1029 (87.4)	61 (43.0)	2208 (100.0)	212 (11.2)	310 (20.6)	510 (100.0)	32 (18.2)	192 (48.4)	0 (0.0)	0 (0.0)	1891 (100.0)
Missing	0 (0.0)	0 (0.0)	0 (0.0)	0 (0.0)	97 (6.5)	0 (0.0)	0 (0.0)	26 (6.5)	0 (0.0)	0 (0.0)	0 (0.0)
Child ASD diagnosis (yes)	15 (1.3)	10 (7.0)	41 (1.9)	826 (43.5)	78 (5.2)	55 (10.8)	41 (23.3)	81 (20.4)	44 (3.2)	61 (6.9)	22 (1.2)
Source of ASD diagnosis											
Measured at study visit using ADOS	0 (0.0)	0 (0.0)	0 (0.0)	0 (0.0)	0 (0.0)	1 (0.2)	0 (0.0)	330 (83.1)	0 (0.0)	61 (6.9)	0 (0.0)
Measured at study visit using other tool	0 (0.0)	0 (0.0)	0 (0.0)	1900 (100.0)^a^	1503 (100.0)	0 (0.0)	174 (98.9)	67 (16.9)	0 (0.0)	0 (0.0)	0 (0.0)
Parent or other caregiver reported at study visits	8 (0.7)	142 (100.0)	0 (0.0)	0 (0.0)	0 (0.0)	509 (99.8)	2 (1.1)	0 (0.0)	1366 (100.0)	0 (0.0)	14 (0.7)
Extracted from medical record or educational record	7 (0.6)	0 (0.0)	2208 (100.0)	0 (0.0)	0 (0.0)	0 (0.0)	0 (0.0)	0 (0.0)	0 (0.0)	0 (0.0)	8 (0.4)
Not applicable	0 (0.0)	0 (0.0)	0 (0.0)	0 (0.0)	0 (0.0)	0 (0.0)	0 (0.0)	0 (0.0)	0 (0.0)	824 (93.1)	1869 (98.8)
Missing	1162 (98.7)	0 (0.0)	0 (0.0)	0 (0.0)	0 (0.0)	0 (0.0)	0 (0.0)	0 (0.0)	0 (0.0)	0 (0.0)	0 (0.0)
Neonatal complications (yes)	425 (36.1)	38 (26.8)	1741 (78.8)	316 (16.6)	116 (7.7)	350 (68.6)	0 (0.0)	23 (5.8)	0 (0.0)	885 (100.0)	70 (3.7)
Missing	39 (3.3)	0 (0.0)	0 (0.0)	1503 (79.1)	0 (0.0)	0 (0.0)	176 (100.0)	374 (94.2)	1366 (100.0)	0 (0.0)	0 (0.0)
Maternal age in years, mean (SD)	27.8 (6.3)	20.9 (2.8)	30.8 (5.1)	30.6 (5.8)	26.8 (5.4)	28.8 (6.4)	25.6 (4.9)	34.1 (4.8)	32.7 (5.2)	29.2 (6.7)	31.5 (5.2)
Missing	0	0	0	0	97	8	0	28	0	0	0
Maternal race											
White	883 (75.0)	6 (4.2)	592 (26.8)	1540 (81.1)	467 (31.1)	243 (47.6)	128 (72.7)	251 (63.2)	1028 (75.3)	545 (61.6)	910 (48.1)
Black	187 (15.9)	0 (0.0)	121 (5.5)	93 (4.9)	936 (62.3)	97 (19.0)	35 (19.9)	23 (5.8)	219 (16.0)	237 (26.8)	70 (3.7)
Asian	41 (3.5)	1 (0.7)	429 (19.4)	144 (7.6)	13 (0.9)	36 (7.1)	0 (0.0)	40 (10.1)	68 (5.0)	15 (1.7)	121 (6.4)
Native Hawaiian or other Pacific islander	0 (0.0)	11 (7.7)	18 (0.8)	6 (0.3)	1 (0.1)	2 (0.4)	0 (0.0)	2 (0.5)	0 (0.0)	0 (0.0)	2 (0.1)
American Indian or Alaska Native	10 (0.8)	32 (22.5)	5 (0.2)	11 (0.6)	1 (0.1)	9 (1.8)	3 (1.7)	3 (0.8)	0 (0.0)	12 (1.4)	5 (0.3)
Multiple race	53 (4.5)	18 (12.7)	372 (16.8)	106 (5.6)	77 (5.1)	70 (13.7)	8 (4.5)	23 (5.8)	43 (3.1)	0 (0.0)	28 (1.5)
Other race	3 (0.3)	73 (51.4)	555 (25.1)	0 (0.0)	6 (0.4)	48 (9.4)	0 (0.0)	13 (3.3)	2 (0.1)	54 (6.1)	210 (11.1)
Missing	0 (0.0)	1 (0.7)	116 (5.3)	0 (0.0)	2 (0.1)	5 (1.0)	2 (1.1)	42 (10.6)	6 (0.4)	22 (2.5)	545 (28.8)
Maternal ethnicity—Hispanic	291 (24.7)	13 (9.2)	781 (35.4)	482 (25.4)	32 (2.1)	106 (20.8)	16 (9.1)	66 (16.6)	108 (7.9)	86 (9.7)	766 (40.5)
Missing	2 (0.2)	100 (70.4)	120 (5.4)	0 (0.0)	9 (0.6)	4 (0.8)	2 (1.1)	40 (10.1)	6 (0.4)	2 (0.2)	277 (14.6)
Maternal education											
High school or less	0 (0.0)	125 (88.0)	219 (9.9)	507 (26.7)	893 (59.4)	198 (38.8)	18 (10.2)	34 (8.6)	116 (8.5)	353 (39.9)	560 (29.6)
Some college	0 (0.0)	16 (11.3)	584 (26.4)	575 (30.3)	138 (9.2)	176 (34.5)	13 (7.4)	110 (27.7)	298 (21.8)	201 (22.7)	204 (10.8)
Bachelor's degree or above	0 (0.0)	1 (0.7)	924 (41.8)	801 (42.2)	470 (31.3)	124 (24.3)	2 (1.1)	223 (56.2)	946 (69.3)	305 (34.5)	830 (43.9)
Missing	1177 (100.0)	0 (0.0)	481 (21.8)	17 (0.9)	2 (0.1)	12 (2.4)	143 (81.3)	30 (7.6)	6 (0.4)	26 (2.9)	297 (15.7)
Parity											
Nulliparous	527 (44.8)	81 (57.0)	663 (30.0)	782 (41.2)	621 (41.3)	0 (0.0)	0 (0.0)	0 (0.0)	662 (48.5)	516 (58.3)	617 (32.6)
1–2	495 (42.1)	57 (40.1)	1295 (58.7)	902 (47.5)	691 (46.0)	0 (0.0)	26 (14.8)	219 (55.2)	645 (47.2)	304 (34.4)	1094 (57.9)
≥ 3	0 (0.0)	4 (2.8)	245 (11.1)	115 (6.1)	191 (12.7)	0 (0.0)	141 (80.1)	53 (13.4)	59 (4.3)	65 (7.3)	180 (9.5)
Missing	155 (13.2)	0 (0.0)	5 (0.2)	101 (5.3)	0 (0.0)	510 (100.0)	9 (5.1)	125 (31.5)	0 (0.0)	0 (0.0)	0 (0.0)
Time of initiation for maternal prenatal health care access											
1st trimester	–	–	2174 (98.5)	1712 (90.1)	–	–	133 (75.6)	116 (29.2)	1366 (100.0)	–	0 (0.0)
2nd trimester	–	–	30 (1.4)	163 (8.6)	–	–	29 (16.5)	16 (4.0)	0 (0.0)	–	1891 (100.0)
3rd trimester	–	–	4 (0.2)	19 (1.0)	–	–	2 (1.1)	3 (0.8)	0 (0.0)	–	0 (0.0)
No access	–	–	0 (0.0)	1 (0.1)	–	–	6 (3.4)	0 (0.0)	0 (0.0)	–	0 (0.0)
Missing	1177 (100.0)	142 (100.0)	0 (0.0)	5 (0.3)	1503 (100.0)	510 (100.0)	6 (3.4)	262 (66.0)	0 (0.0)	885 (100.0)	0 (0.0)
Maternal prenatal smoking (yes)	190 (16.1)	9 (6.3)	97 (4.4)	186 (9.8)	151 (10.0)	67 (13.1)	57 (32.4)	22 (5.5)	132 (9.7)	220 (24.9)	238 (12.6)
Missing	0 (0.0)	0 (0.0)	183 (8.3)	224 (11.8)	1 (0.1)	2 (0.4)	1 (0.6)	3 (0.8)	7 (0.5)	18 (2.0)	0 (0.0)
Maternal BMI (kg/m^2^), mean (SD)	25.7 (6.2)	23.9 (5.8)	27.6 (6.5)	26.1 (6.2)	27.5 (7.5)	28.3 (7.6)	29.0 (10.3)	27.3 (6.9)	24.7 (5.2)	26.6 (7.1)	25.8 (5.5)
Missing	0	100	23	71	5	14	4	127	8	32	60
Maternal diabetes	50 (4.2)	0 (0.0)	273 (12.4)	183 (9.6)	80 (5.3)	0 (0.0)	8 (4.5)	54 (13.6)	78 (5.7)	841 (95.0)	233 (12.3)
Missing	0 (0.0)	142 (100.0)	8 (0.4)	71 (3.7)	60 (4.0)	510 (100.0)	7 (4.0)	114 (28.7)	9 (0.7)	44 (5.0)	0 (0.0)
Maternal high blood pressure	139 (11.8)	0 (0.0)	275 (12.5)	195 (10.3)	141 (9.4)	97 (19.0)	20 (11.4)	37 (9.3)	158 (11.6)	861 (97.3)	210 (11.1)
Missing	33 (2.8)	142 (100.0)	0 (0.0)	1705 (89.7)	73 (4.9)	413 (81.0)	6 (3.4)	360 (90.7)	33 (2.4)	24 (2.7)	1681 (88.9)
Maternal history of any psychiatric disorders	204 (17.3)	0 (0.0)	1203 (54.5)	871 (45.8)	31 (2.1)	210 (41.2)	119 (67.6)	38 (9.6)	140 (10.2)	0 (0.0)	216 (11.4)
Missing	29 (2.5)	142 (100.0)	0 (0.0)	561 (29.5)	293 (19.5)	0 (0.0)	0 (0.0)	271 (68.3)	207 (15.2)	885 (100.0)	0 (0.0)
Maternal alcohol use	76 (6.5)	2 (1.4)	273 (12.4)	391 (20.6)	121 (8.1)	13 (2.5)	19 (10.8)	31 (7.8)	859 (62.9)	0 (0.0)	51 (2.7)
Missing	0 (0.0)	1 (0.7)	257 (11.6)	245 (12.9)	1 (0.1)	1 (0.2)	5 (2.8)	277 (69.8)	86 (6.3)	885 (100.0)	34 (1.8)
Maternal folic acid supplementation	471 (40.0)	0 (0.0)	1107 (50.1)	206 (10.8)	1239 (82.4)	0 (0.0)	0 (0.0)	30 (7.6)	155 (11.3)	0 (0.0)	0 (0.0)
Missing	0 (0.0)	142 (100.0)	1056 (47.8)	235 (12.4)	181 (12.0)	510 (100.0)	176 (100.0)	122 (30.7)	20 (1.5)	885 (100.0)	1891 (100.0)
Maternal other vitamin and mineral supplements	1174 (99.7)	0 (0.0)	1107 (50.1)	1525 (80.3)	1392 (92.6)	0 (0.0)	0 (0.0)	259 (65.2)	1301 (95.2)	0 (0.0)	1827 (96.6)
Missing	0 (0.0)	142 (100.0)	1056 (47.8)	249 (13.1)	33 (2.2)	510 (100.0)	176 (100.0)	125 (31.5)	53 (3.9)	885 (100.0)	0 (0.0)

*Note*: Data shown are *n* (%) except where indicated.

Abbreviations: ADI‐R, autism diagnostic interview‐revised; ADOS, autism diagnostic observation schedule.

^a^ Cohort 4 utilized both ADI‐R and ADOS to obtain ASD diagnosis.

**TABLE 2:** Participant characteristics by cohorts included in analysis of Social Responsiveness Scale (SRS)

	Cohort 2	Cohort 4	Cohort 8	Cohort 9	Cohort 10	Cohort 12	Cohort 13
*n*	38	138	219	403	866	907	111
Type of study population	General population *n* (%)	Oversampled for ASD cases *n* (%)	Oversampled for ASD risk (familial ASD) *n* (%)	General population *n* (%)	Oversampled for ASD risk (preterm birth) *n* (%)	General population *n* (%)	General population *n* (%)
Child SRS T‐score, mean (SD)	53.2 (8.4)	60.2 (16.8)	53.2 (13.0)	47.1 (8.5)	54.5 (14.9)	45.1 (6.1)	52.9 (8.7)
Age at child SRS test in months, mean (SD)	93.8 (26.6)	150.1 (31.9)	38.1 (4.5)	209.3 (4.8)	119.0 (8.0)	43.1 (11.6)	58.1 (11.2)
Missing observations	0	0	37	4	0	0	0
Version of SRS test							
SRS™	0 (0.0)	0 (0.0)	124 (56.6)	0 (0.0)	0 (0.0)	0 (0.0)	0 (0.0)
SRS – 2 Preschool Form™	4 (10.5)	0 (0.0)	40 (18.3)	0 (0.0)	0 (0.0)	750 (82.7)	111 (100.0)
SRS – 2 School Form™	34 (89.5)	138 (100.0)	0 (0.0)	403 (100.0)	0 (0.0)	157 (17.3)	0 (0.0)
Missing	0 (0.0)	0 (0.0)	55 (25.1)	0 (0.0)	866 (100.0)	0 (0.0)	0 (0.0)
Maternal prenatal smoking (yes)	3 (7.9)	12 (8.7)	15 (6.8)	39 (9.7)	214 (24.7)	44 (4.9)	11 (9.9)
Missing	0 (0.0)	3 (2.2)	3 (1.4)	1 (0.2)	16 (1.8)	41 (4.5)	0 (0.0)
Child female	19 (50)	34 (24.6)	92 (42.0)	213 (52.9)	426 (49.2)	455 (50.2)	55 (49.5)
Missing	0 (0.0)	0 (0.0)	14 (6.4)	0 (0.0)	0 (0.0)	0 (0.0)	0 (0.0)
Year of birth of child	0 (0.0)	0 (0.0)	0 (0.0)	0 (0.0)	0 (0.0)	0 (0.0)	0 (0.0)
Before 1991	0 (0.0)	0 (0.0)	0 (0.0)	0 (0.0)	0 (0.0)	0 (0.0)	0 (0.0)
1991–2000	0 (0.0)	7 (5.1)	0 (0.0)	259 (64.3)	0 (0.0)	0 (0.0)	0 (0.0)
2001–2010	22 (57.9)	131 (94.9)	50 (22.8)	144 (35.7)	866 (100.0)	167 (18.4)	73 (65.8)
After 2010	16 (42.1)	0 (0.0)	152 (70.4)	0 (0.0)	0 (0.0)	714 (82.4)	38 (34.2)
Missing	0 (0.0)	0 (0.0)	14 (6.5)	0 (0.0)	0 (0.0)	0 (0.0)	0 (0.0)
Neonatal complications (yes)	8 (21.1)	28 (20.3)	7 (3.2)	0 (0.0)	866 (100.0)	74 (8.2)	0 (0.0)
Missing	0 (0.0)	99 (71.7)	212 (96.8)	403 (100.0)	0 (0.0)	0 (0.0)	111 (100.0)
Maternal age in years, mean (SD)	21.0 (2.7)	31.1 (6.1)	34.0 (4.8)	33.0 (5.1)	29.3 (6.7)	31.7 (4.7)	25.9 (5.2)
Missing	0	0	14	0	0	0	0
Maternal race							
White	3 (7.9)	117 (84.8)	128 (58.4)	307 (76.2)	535 (61.8)	837 (92.3)	92 (82.9)
Black	0 (0.0)	3 (2.2)	16 (7.3)	58 (14.4)	235 (27.1)	0 (0.0)	14 (12.6)
Asian	0 (0.0)	7 (5.1)	24 (11.0)	26 (6.5)	15 (1.7)	5 (0.6)	1 (0.9)
Native Hawaiian or other Pacific islander	5 (13.2)	1 (0.7)	1 (0.5)	0 (0.0)	0 (0.0)	1 (0.1)	0 (0.0)
American Indian or Alaska Native	7 (18.4)	2 (1.4)	3 (1.4)	0 (0.0)	11 (1.3)	2 (0.2)	0 (0.0)
Multiple race	3 (7.9)	8 (5.8)	15 (6.8)	12 (3.0)	0 (0.0)	9 (1.0)	2 (1.8)
Other race	20 (52.6)	0 (0.0)	10 (4.6)	0 (0.0)	50 (5.8)	0 (0.0)	2 (1.8)
Missing	0 (0.0)	0 (0.0)	22 (10.0)	0 (0.0)	20 (2.3)	53 (5.8)	0 (0.0)
Maternal ethnicity—Hispanic	5 (13.2)	31 (22.5)	32 (14.6)	33 (8.2)	79 (9.1)	20 (2.2)	16 (14.4)
Missing	26 (68.4)	0 (0.0)	18 (8.2)	0 (0.0)	2 (0.2)	53 (5.8)	1 (0.9)
Maternal education							
High school or less	34 (89.5)	27 (19.6)	23 (10.5)	30 (7.4)	344 (39.7)	71 (7.8)	41 (36.9)
Some college	4 (10.5)	31 (22.5)	59 (26.9)	69 (17.1)	199 (23.0)	169 (18.6)	40 (36.0)
Bachelor's degree or above	0 (0.0)	79 (57.2)	121 (55.3)	304 (75.4)	300 (34.6)	625 (68.9)	28 (25.2)
Missing	0 (0.0)	1 (0.7)	16 (7.3)	0 (0.0)	23 (2.7)	42 (4.6)	2 (1.8)
Parity							
Nulliparous	23 (60.5)	64 (46.4)	0 (0.0)	191 (47.4)	504 (58.2)	390 (43.0)	51 (45.9)
1–2	15 (39.5)	64 (46.4)	132 (60.3)	196 (48.6)	297 (34.2)	436 (48.1)	37 (33.3)
≥ 3	0 (0.0)	9 (6.5)	29 (13.2)	16 (4.0)	65 (7.5)	70 (7.7)	0 (0.0)
Missing	0 (0.0)	1 (0.7)	58 (26.5)	0 (0.0)	0 (0.0)	11 (1.2)	23 (20.7)
Time of initiation for maternal prenatal health care access							
1st trimester	–	126 (91.3)	35 (16.0)	403 (100.0)	–	793 (87.4)	–
2nd trimester	–	12 (8.7)	3 (1.4)	0 (0.0)	–	16 (1.8)	–
3rd trimester	–	0 (0.0)	2 (0.9)	0 (0.0)	–	1 (0.1)	–
No access	–	0 (0.0)	0 (0.0)	0 (0.0)	–	0 (0.0)	–
Missing	38 (100.0)	0 (0.0)	179 (81.7)	0 (0.0)	866 (100.0)	97 (10.7)	111 (100.0)
Maternal BMI (kg/m^2^), mean (SD)	24.2 (5.2)	25.7 (5.8)	28.5 (7.6)	24.6 (5.3)	26.6 (7.1)	25.8 (5.3)	27.6 (7.8)
Missing	33	4	60	0	28	12	1
Maternal diabetes	0 (0.0)	17 (12.3)	27 (12.3)	20 (5.0)	821 (94.8)	68 (7.5)	3 (2.7)
Missing	38 (100.0)	1 (0.7)	55 (25.1)	4 (1.0)	45 (5.2)	111 (12.2)	0 (0.0)
Maternal high blood pressure	0 (0.0)	10 (7.2)	18 (8.2)	43 (10.7)	842 (97.2)	101 (11.1)	5 (4.5)
Missing	38 (100.0)	128 (92.8)	201 (91.8)	12 (3.0)	24 (2.8)	225 (24.8)	106 (95.5)
Maternal history of any psychiatric disorders	0 (0.0)	70 (50.7)	33 (15.1)	42 (10.4)	0 (0.0)	148 (16.3)	0 (0.0)
Missing	38 (100.0)	20 (14.5)	108 (49.3)	56 (13.9)	866 (100.0)	12 (1.3)	111 (100.0)
Maternal alcohol use	1 (2.6)	35 (25.4)	20 (9.1)	248 (61.5)	0 (0.0)	147 (16.2)	0 (0.0)
Missing	0 (0.0)	4 (2.9)	119 (54.3)	19 (4.7)	866 (100.0)	41 (4.5)	0 (0.0)
Maternal folic acid supplementation	0 (0.0)	19 (13.8)	17 (7.8)	48 (11.9)	0 (0.0)	774 (85.3)	0 (0.0)
Missing	38 (100.0)	3 (2.2)	56 (25.6)	4 (1.0)	866 (100.0)	71 (7.8)	111 (100.0)
Maternal other vitamin and mineral supplements	0 (0.0)	115 (83.3)	155 (70.8)	391 (97.0)	0 (0.0)	773 (85.2)	0 (0.0)
Missing	38 (100.0)	5 (3.6)	57 (26.0)	10 (2.5)	866 (100.0)	72 (7.9)	111 (100.0)

*Note*: Data shown are *n* (%) except where indicated.

Abbreviations: ASD, autism spectrum disorder; SD, standard deviation.

#### ASD diagnosis

ASD is defined as a developmental disability involving four domains: social reciprocal relationships, communication skills, repetitive behaviors or restricted interests, and sensory sensitivities (American Psychiatric Association, 2013). Sources of data on ASD diagnosis included established standardized instruments, the Autism Diagnostic Observation Schedule (ADOS; used by three cohorts) (Lord et al., 2000) and/or other instruments (four cohorts); parental or other caregiver report of an ASD diagnosis (five cohorts); and/or a diagnosis extracted from medical or educational records (three cohorts).

#### SRS T‐score

The SRS is a 65‐item rating scale that provides a continuous measure of the severity of social impairments in both ASD‐affected and general populations based on responses from the primary caregiver. Each item is rated from 0 (never true) to 3 (almost always true). Total raw scores range from 0 to 195. Forms used were preschool (2 cohorts), school‐aged (2 cohorts), both (2 cohorts), or unknown (1 cohort). The school‐aged form was used for children 4–18 years of age and preschool forms for ages 2.5–4.5 years. The intentional overlap in age ranges allowed for variability in developmental stage. Sex‐normed T‐scores were calculated to facilitate clinical utility (Constantino & Gruber, 2012). We also dichotomized SRS scores (≥66 vs. <66) to distinguish mild from moderate/severe symptoms typically consistent with an ASD diagnosis (Constantino & Gruber, 2005, 2012).

#### Maternal smoking

The principal exposure of interest was maternal active smoking of standard cigarettes (not e‐cigarettes) at any time point during pregnancy or in the 6 months prior to conception. All studies collected prenatal maternal smoking, and two additionally had pre‐conceptional maternal smoking information through personal self‐report (e.g., interviews or surveys), typically during pregnancy or around the time of birth or shortly thereafter, or in the case–control study, during early childhood.

#### Covariate selection

Our approach to model‐building relied on causal methods exclusively. We constructed a directed acyclic graph (DAG) based on reviewing the literature and considering a wide swath of variables causally or non‐causally associated with the exposure, the outcome, or other covariates (Supplemental Figure 1). After extensive discussion among authors, we reached a final consensus DAG. We identified all backdoor paths in this DAG, and then, for each cohort we identified at least one set of sufficient variables to block as many backdoor paths as possible. During the analysis phase, no variables were dropped or added. Thus, selection of covariates was governed entirely by the underlying causal model represented by the DAG.

After excluding variables previously found to be weakly associated with outcome or exposure, covariates initially considered included maternal age; education; history of psychiatric disorders; Type 1, Type 2, or gestational diabetes; high blood pressure or pre‐eclampsia; alcohol use; folic acid or other vitamin supplementation; parity; and trimester of initiation of prenatal care as an indicator of access to healthcare; along with neonatal complications (Table 1). Diabetes was considered a confounder as it shares a common cause with smoking, namely maternal education/socioeconomic status (SES) operating through health literacy and/or access to quality health care. The same set of covariates was used for both ASD diagnosis and SRS score. Because not all covariates were collected by every cohort, we identified cohort‐specific sets of covariates, each sufficient to adjust for confounding of the maternal smoking associations with outcomes (Supplemental Table 1, cohort‐specific adjustment sets).

### Statistical analysis

We conducted a disseminated meta‐analysis using results from standardized cohort‐specific analyses (Jacobson et al., 2018). The ECHO Data Analysis Center provides cohorts with (1) a data dictionary with variable names and definitions, and (2) statistical code to perform a standardized analysis based on their cohort's population and dataset. This disseminated meta‐analysis was unique in customizing each cohort's code according to availability of key covariates. Participant characteristics were compared across cohorts. To account for unbalanced adjustments across cohorts, we derived a propensity score of maternal smoking conditioning on cohort specific covariates, then generated stabilized inverse probability weights (Rosenbaum & Rubin, 1983). Covariate balance after weighting was checked by comparing the difference in covariates between maternal smoking and nonsmoking groups. We pooled estimates of beta coefficients from 11 cohorts for ASD, and separately, seven cohorts for SRS T‐scores. Logistic regression models and linear regression models with inverse probability weighting were used, respectively, to obtain the OR or standardized mean difference (SMD) with a 95% CI for corresponding binary and continuous outcomes, comparing maternal preconception/prenatal smoking versus nonsmoking groups. We pooled estimates using random effects for cohort. Heterogeneity was quantified using the Cochran's *Q* test (a chi‐squared test; Higgins, 2003) and stratified analyses (such as general and non‐general population cohorts, which oversampled cases or were restricted to subgroups with elevated ASD risk). The Cochran's *Q* test, which is calculated as the weighted sum of squared differences between individual study effects and the pooled effect across studies, is distributed as a chi‐squared statistic with *k* (number of studies) minus 1 degree of freedom.

Subset analyses were conducted to assess heterogeneity according to study design or source populations, including general population cohorts and special cohorts, which were further divided as (1) restricted to preterm births, or (2) oversampled for ASD (case–control) or high familial risk of ASD. Further sensitivity analyses were conducted. Cohorts having 4 or fewer cases in either exposure group (*n* = 3 cohorts) were dropped. Preterm birth cohorts (*n* = 2 cohorts) were dropped for the following reasons: (1) conditioning on a factor that could be an intermediate on a causal pathway produces biased estimates of the total effect; (2) even if preterm birth is not an intermediate, effect measures in preterm cohorts can differ from effects in mixed, predominantly full‐term cohorts because of their skewed distribution of high‐risk conditions (Snowden & Basso, 2018); and (3) the lack of opportunity for a third trimester exposure can introduce bias if, as is true of preterm delivery, exposure opportunity is associated with the outcome (Schieve et al., 2016). Preterm births in other cohorts, that is, mixed cohorts with a preponderance of children born full‐term, were never excluded from the analysis.

This sensitivity analysis then included six cohorts for the outcome of ASD diagnosis. To examine robustness and sensitivity of pooled estimates to each individual cohort estimate, we used the “leave‐one out” approach. The leave‐one‐out approach utilizes several statistics or indicators of influence, including (1) rstudent (externally standardized residual); (2) dffits, which indicates how many standard deviations the predicted (average) effect for the (i)th study changes after excluding the (i)th study from the meta‐analysis; (3) Mahalanobis distance (cook.d), defined as the distance between the entire set of predicted values once with the (i)th study included and once with the (i)th study excluded from the meta‐analysis; (4) covariance ratio, (cov.r) defined as the determinant of the variance–covariance matrix of the parameter estimates based on the dataset with the (i)th study removed divided by the determinant of the variance–covariance matrix of the parameter estimates based on the complete dataset (a value below 1 indicates that removal of the (i)th study yields more precise estimates of the model coefficients); (5) tau2.del, the leave‐one‐out amount of (residual) heterogeneity based on the dataset with the (i)th study removed; (6) the leave‐one‐out test statistic (QE.del) for the test of (residual) heterogeneity, which is the value of the test statistic for (residual) heterogeneity calculated based on the dataset with the (i)th study removed; (7) the diagonal elements of the hat matrix (hat); and (8) the weights (in %) given to the observed effects or outcomes during the meta‐analysis. All statistical analyses were performed using R software (R Core Team, 2018). We used the metagen function within the meta package to pool the estimates and the forest function to generate forest plots (Schwarzer, 2007).

## RESULTS

### Cohort characteristics

Study designs, sample sizes, and descriptive data on exposure and covariates are presented for analyses of ASD (11 cohorts, Table 1) and SRS (seven cohorts, Table 2). Sample sizes for ASD cohorts ranged from 142 to 2208. Due to the male preponderance of ASD, studies that oversampled for ASD cases or restricted to enhanced ASD risk families were heavily weighted for males. Besides these study designs, three general population cohorts (including the two smallest) also had higher than expected diagnoses of ASD (Maenner et al., 2021). Prevalence of pregnancy complications ranged from 3.7% to 100%, with two preterm birth cohorts having 100% by design.

Cohorts varied in maternal age, education, and parity. Racial and ethnic composition was highly diverse across studies. Among cohorts with relevant information, most women initiated prenatal care during the first or second trimester. The prevalence of vitamin or mineral supplementation was high in all cohorts reporting this information, and maternal psychiatric conditions varied widely in prevalence (2.1–67.6%) as did diabetes, hypertension, and alcohol use. The prevalence of maternal smoking during the preconception and prenatal period ranged from 4.4% to 32.4%.

Sample sizes for the SRS analyses ranged from 38 to 907 participants (Table 2). Both maternal and child characteristics were similar to those in the ASD diagnosis analysis, including child's birth year, gender, and neonatal complications, as well as maternal mean age, race, ethnicity, parity, health conditions, health care access, use of alcohol, smoking, and use of prenatal supplements. Mean standardized child SRS T‐scores ranged across cohorts from 45.1 to 60.2 and were higher in cohorts enriched for ASD or ASD risk. The SRS, 2nd edition (SRS‐2) Preschool Form was predominantly used by two cohorts with average ages of 43.1–58.1 months, and the SRS‐2 School Form was used by three cohorts with average ages of 93.8–209.3 months. One cohort mainly used the SRS form; another was missing information on the form used but reported a mean age of 119 months.

### Preconception/prenatal maternal smoking and ASD risk

**FIGURE 1:**
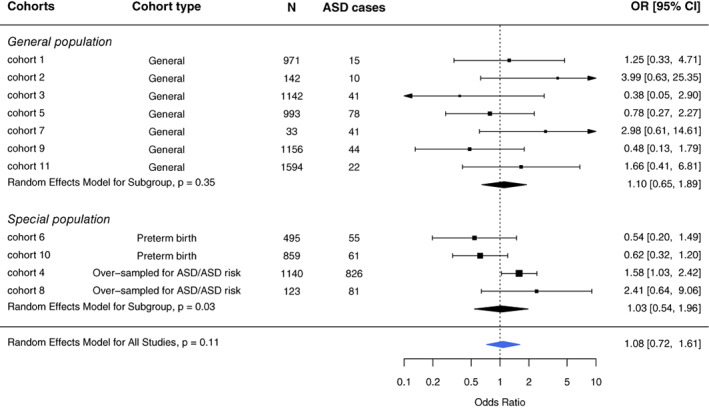
Association between maternal prenatal smoking and the risk of autism spectrum disorder (ASD) based on random effects model overall and by cohort type. Participants with non‐missing values in cohort‐specific sufficient adjustment sets were included in the disseminated analyses and hence the meta‐analysis. The sample sizes listed represent the total sample included in the meta‐analysis; these may differ from those listed in Table 1 because of missing covariate data

**FIGURE 2:**
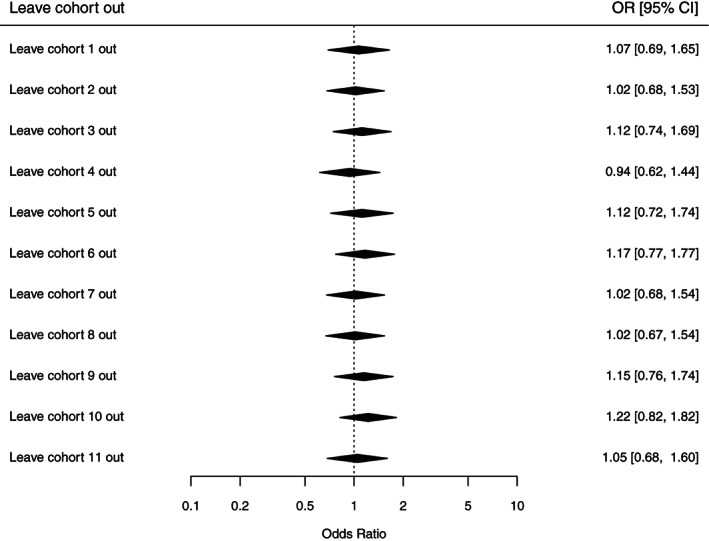
Odds ratio plots from the leave‐one‐out analyses for autism spectrum disorder (ASD)

**FIGURE 3:**
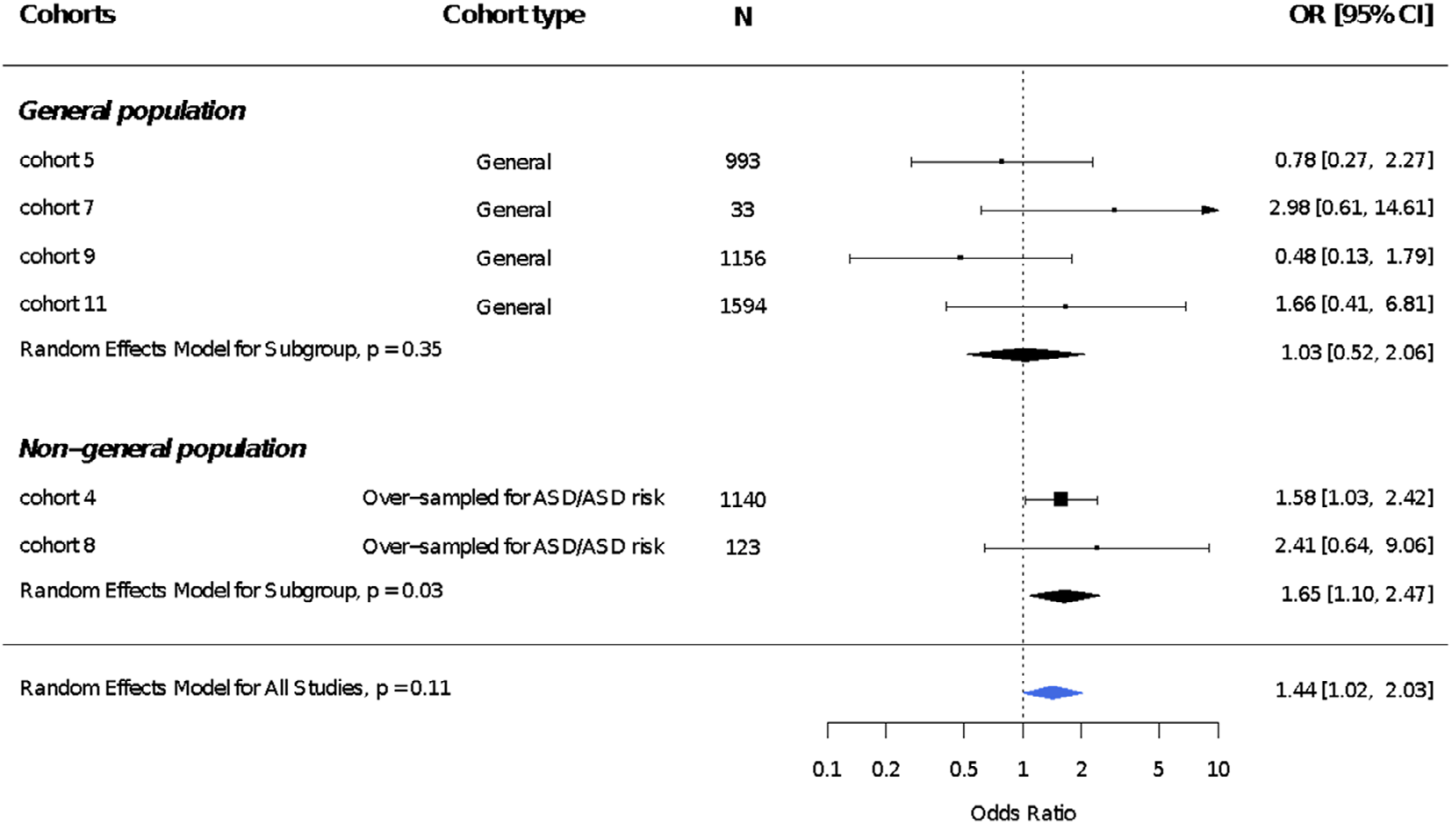
Association between maternal prenatal smoking and the risk of autism spectrum disorder (ASD) based on random effects model overall and by cohort type dropping preterm birth cohorts and cohorts with 4 or fewer ASD cases in either the exposed or unexposed group. The sample sizes listed represent the total sample included in the meta‐analysis; these may differ from those listed in Table 1 because of missing data

Effect estimates of 11 cohorts (8648 participants) were pooled to examine the relationship of preconception and prenatal maternal smoking and ASD diagnosis. We did not observe an association after pooling the estimates across all 11 cohorts, with an OR of 1.08 (95% CI, 0.72–1.61; Figure 1). The leave‐one‐out analysis was performed with eight standard statistics as discussed in the Methods section (Figure 2) and showed very little influence of single cohorts. Based on the diagnostic plots (Supplemental Figures), no highly influential cohorts were found, but cohorts 4 and 10 were moderately more influential than the other cohorts based on their higher cook.d, and lower tau2.del and QE.del values, with the two cohorts influencing the meta‐analysis in opposite directions (Figure 2). In subset analyses, the ORs were similar across the two source populations (general and special). However, among the special population cohorts, preterm birth cohorts showed estimated OR's below one, whereas autism‐enriched risk cohorts had OR estimates above one (Figure 1). Given the numerous ways in which the preterm cohorts (*n* = 2) could produce biased estimates (see Methods), we removed these from our analyses to improve validity, as well as cohorts with four or fewer ASD cases in either the exposed or unexposed groups (*n* = 3) to achieve a more reliable pooled OR. After those exclusions, the association between maternal preconception/prenatal smoking and risk of ASD diagnosis (Figure 3) increased to an OR of 1.44 (95% CI, 1.02–2.03).

### Preconception/prenatal maternal tobacco smoking and ASD‐related quantitative trait scores

**FIGURE 4:**
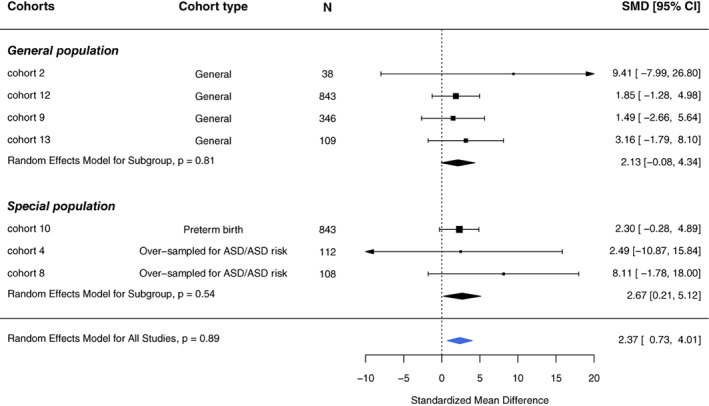
Association between maternal prenatal smoking and Social Responsiveness Scale (SRS) T‐scores based on random effects models overall and by cohort type. Participants with non‐missing values in cohort‐specific sufficient adjustment sets were included in the disseminated analyses and hence the meta‐analysis. The sample sizes listed represent the total sample included in the meta‐analysis; these may differ from those listed in Table 2 because of missing data

**FIGURE 5:**
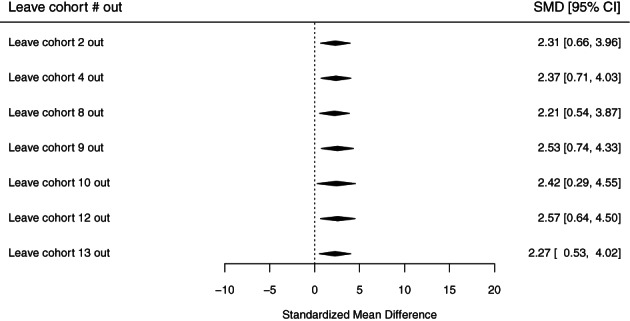
Odds ratio plots from the leave‐one‐out analyses for the dichotomized (T‐score ≥ 66 vs. <66) social responsiveness scale (SRS)

A total of 2399 participants from seven cohorts were included in our meta‐analyses of preconception or prenatal maternal smoking and ASD‐related quantitative traits, assessed as SRS standardized *T*‐scores. Compared with children whose mothers did not report smoking in the preconception or prenatal periods, children whose mothers did report preconception/prenatal smoking scored 2.37 (95% CI, 0.73–4.01) points higher on the SRS (Figure 4). This result represents approximately a one‐fifth standard deviation increase in SRS T‐score, indicative of greater social impairment. Results were consistent across cohorts and by source population (general population: SMD, 2.13 [95% CI, −0.08‐4.34]; non‐general population: SMD, 2.67 [95% CI, 0.21–5.12]; Figure 3). Dichotomizing the *T*‐score, we observed an increased odds of having moderate to severe symptoms in children of women who smoked during the preconception/prenatal period (OR, 1.62; 95% CI, 0.87–3.01; Supplemental Figure 1). Results were consistent across cohorts and by source population (general population: OR, 2.53 [95% CI, 0.92–6.95]; non‐general population: OR, 1.31 [95% CI, 0.61–2.78]; Supplemental Figure 1). The leave‐one‐out SRS analysis was performed with eight standard statistics as discussed in the section 2 (Figure 5). Based on the diagnostic plots, no influential cohorts were found due to similar tau2 values, suggesting little heterogeneity after removing any cohort.

## DISCUSSION

In this aggregate meta‐analysis, we found that, contrary to expectation, maternal preconception or prenatal tobacco smoking was not associated with the risk for ASD. However, after excluding studies with small cell sizes and other studies that conditioned on a potential intermediate variable (preterm birth)—a design which reduced opportunity for exposure and does not correctly estimate the total effect—we observed a 1.44‐fold increased odds of ASD among children of smoking mothers. Further, autism‐related traits, assessed with the SRS, were associated with prenatal/preconception maternal smoking. Thus, these meta‐analyses suggest modest associations of maternal preconception/prenatal tobacco use with both poorer social communication skills and elevated risk for ASD. These findings add to the numerous other adverse outcomes associated with maternal smoking during gestation: higher risks for ectopic pregnancy (Gaskins et al., 2018; Handler et al., 1989; Stergachis et al., 1991), fetal loss (Flenady et al., 2011; Marufu et al., 2015; Pineles et al., 2014; Pineles et al., 2016), preterm delivery (Liu et al., 2020; Soneji & Beltrán‐Sánchez, 2019), and lower birthweight (Blatt et al., 2015; Günther et al., 2021; Pereira et al., 2017; Tayie & Powell, 2012), as well as childhood outcomes of asthma (Harju et al., 2016; McEvoy & Spindel, 2017; Neuman et al., 2012) and attention deficit‐hyperactivity disorder (Huang et al., 2018; Langley et al., 2005; Sourander et al., 2019).

In contrast with previous meta‐analyses of the literature, (1) we took a systematic approach to adjustment for confounders based in causal theory and prior knowledge; (2) all cohorts were actively enrolled, reducing under‐ascertainment of ASD that characterizes many administrative databases; (3) the study populations were quite diverse in terms of socioeconomic, racial, and ethnic distributions, supporting greater generalizability; and (4) the meta‐analyzed data were not based on published reports but on disseminated analyses using a harmonized approach to exposure and covariates.

Mechanisms by which prenatal tobacco smoke exposure might disrupt fetal brain development include both direct toxicity as well as effects on placental function. The most important components of tobacco smoke that are hazardous for the fetus are nicotine and carbon monoxide (Ekblad et al., 2015). Carbon monoxide crosses the placenta and binds to hemoglobin, potentially lowering oxygen delivery to fetal tissues, and preterm infants exposed prenatally to tobacco smoke have lower cerebral oxygen saturation (Verhagen et al., 2011). Nicotine moves freely from maternal blood to fetal tissues, and levels of cotinine, the predominant metabolite of nicotine, are higher in fetal serum than in maternal serum (Jauniaux et al., 1999). Chronic exposure to nicotine alters nicotinic acetylcholine receptors, which play critical roles in fetal brain development (Role & Berg, 1996), and reduces the turnover of two neurotransmitters in the brain—serotonin and dopamine (Muneoka et al., 1997). In addition, prenatal tobacco exposure is associated with epigenetic changes in fetal tissue, placenta, and umbilical cord blood (Chatterton et al., 2017; Ivorra et al., 2015; Martin & Fry, 2018; Paquette et al., 2013) and thus could program for dysregulated neuroendocrine or neuroimmune systems, manifesting as abnormalities of neurocognition or neurobehavior (Vaiserman & Koliada, 2017).

Our findings appear to contradict conclusions drawn in an analysis of a European birth cohort, which concluded that residual confounding accounted for any association between maternal smoking during pregnancy and risk for ASD (Caramaschi et al., 2018). However, the multiple models presented by these authors used different samples when adding covariates, which raises the question: are the effect measures for maternal smoking comparable across models? Under certain conditions, a complete case analysis does not introduce bias in a logistic regression (Bartlett et al., 2015), but bias is expected when missingness is associated with both the exposure and outcome. For the study population of Caramaschi and colleagues, missingness of paternal smoking and the covariates representing socioeconomic status may well have been associated with the joint distribution of maternal smoking and child's ASD outcome, which would have produced bias in the estimated exposure‐outcome association. Selection bias arising from differential missingness is a plausible explanation for the changes they observed in smoking ORs across models. The high proportion of apparent missingness in their key covariates—leading to loss of up to 39% of the cohort, raises further concern about validity of results. Additionally, the high correlation of maternal and paternal smoking demands creative approaches in order to reliably distinguish the independent contributions of either one. Caramaschi and co‐authors assumed that paternal smoking served as a negative control and would at best be a weak contributor to ASD risk. However, research on male smoking in relation to sperm quality, germ line mutations, and epigenetics of spermatozoa suggests strong environmental influences with inter‐ and trans‐generational impacts (Donkin & Barrès, 2018).

A limitation of the current analysis and of most other studies is the use of self‐reported smoking information. Biomarkers have revealed that approximately 25% of pregnant smokers do not report their smoking (Moore et al., 2019; Shipton et al., 2009; Swamy et al., 2011). Misclassifying smokers as nonsmokers—if unrelated to their child's outcome or social skills, or if more likely by parents or caregivers of affected children—would bias results toward the null. All cohorts except one collected smoking information prospectively, prior to the child's diagnosis, hence differential misclassification would likely be limited to the one exception (Cohort 4), which was among the larger studies in this meta‐analysis. A further limitation in the exposure assessment was use of a single dichotomous indicator of smoking in preconception (6 months prior to conception) and prenatal periods. Analysis of timing and frequency/quantity of tobacco use, as well as second‐hand smoke exposure (e.g., from other household members), would provide a more refined understanding of the relationship between preconception/prenatal tobacco smoke exposure and child's risk for ASD.

Additionally, not all cohorts in this study used gold standard instruments for diagnosing ASD. Parental report of ASD diagnoses may inflate prevalence estimates. For example, estimates from the National Health Interview Survey (NHIS), which uses parental reports, are consistently higher than those from the Centers for Disease Control and Prevention's Autism and Developmental Disabilities Monitoring network, in which ASD diagnoses are based on expert review of medical and educational records (Xu et al., 2018). In the 2007 NHIS, parents reported that a high proportion of children with ASD lost their diagnoses over time (Kogan et al., 2009), whereas longitudinal autism research finds loss of diagnosis to be far less common (Moulton et al., 2016; Pierce et al., 2019). Misclassification of outcome, if unrelated to smoking, may have biased toward the null and reduced power to detect associations.

Underdiagnosis of ASD in families with lower socioeconomic circumstances, who are more likely to smoke during pregnancy and have less access to healthcare, may have led to bias toward the null. Although the majority of cohorts controlled for either education or an indicator of health care access, a few did not. Bias away from the null could occur if ASD‐associated genes also predispose individuals to addictive behaviors, such as smoking. The majority of cohorts adjusted for maternal or family history of psychiatric conditions, reducing this problem; moreover, the three cohorts that were exceptions were excluded in the sensitivity analyses due to small cell sizes, suggesting higher validity of the final analyses. Two cohorts adjusted for maternal hypertension, which may have been inappropriate if it is on a causal pathway; however, the relationship of smoking to maternal hypertension appears to be complex and any impact of the adjustment on the final results is unclear.

Paternal smoking was not addressed in this analysis and given both the strong correlation with maternal smoking and paternal influences on ASD, for example, age, lifestyle (Oldereid et al., 2018), and potentially epigenetics (Flashner et al., 2013), additional investigation of the role of paternal smoking is warranted. However, precisely because of the strong correlation of paternal and maternal smoking, it will be challenging to reliably distinguish independent contributions of each without an exceptionally large study population. Confounding from post‐natal exposures also cannot be excluded, given the high correlation between prenatal and postnatal smoking. Nevertheless, the pathogenesis of ASD begins early in life and further research is needed to distinguish the critical time windows.

The stronger and more consistent associations of maternal prenatal tobacco use with higher SRS scores as compared to the association with dichotomized ASD diagnosis could very well be due to the detection of milder social and communication impairments by the SRS. However, our analyses for binary SRS T‐score had a different set of cohorts than the analyses for ASD diagnosis, and SRS measures social impairments, not ASD per se. By definition, it is not a diagnostic instrument. Interestingly, results from Caramaschi et al. (2018) similarly show consistent results for social and communication disorders, but not for ASD. One limitation of the SRS‐T score is the potential for rater bias, which could arise if smokers and nonsmokers report their children's behaviors differently. Secondly, while scores above the T‐score cutoff of 66 are generally consistent with moderate to severe impairment, an ASD diagnosis is more likely when symptoms are severe. Third, SRS scores can be influenced by symptoms not specific to ASD (Frazier et al., 2014; Hus et al., 2013), such as attention‐deficit/hyperactivity disorder (ADHD) (Constantino & Gruber, 2012; Reiersen et al., 2007) or cognitive deficits, both of which have been linked, albeit inconsistently (similar to ASD), with prenatal smoking (Dong et al., 2018; Gustavson et al., 2017). Nonetheless, previous work supports convergent validity of the SRS with gold‐standard ASD measures, the Autism Diagnostic Interview‐Revised (ADI‐R) and ADOS, although those with commonly co‐occurring conditions do not score as high on the SRS as those with ASD (Constantino & Frazier, 2013; Constantino & Gruber, 2012).

The observed inverse association between preconception/prenatal maternal tobacco smoking and ASD in cohorts restricted to children born before their third trimester was unexpected and could reflect error due to chance. However, if the third trimester is a critical window in which tobacco smoke may interfere with neurodevelopment, then cohorts of individuals born preterm, who are selected for not having a third trimester—will experience a truncated window for exposure in a vulnerable period. Of potential relevance is the body of research that has examined timing of air pollution exposures, which share hundreds of compounds with tobacco smoke. Notably, many air pollution studies (Chun et al., 2020; Raz et al., 2015; Volk et al., 2013) but not all (Jo et al., 2019) observed the greatest increase in ASD risk with third trimester exposures.

Other possible explanations for the finding from preterm births deserve consideration. For instance, intrauterine infections are associated with preterm labor and delivery, and maternal immune activation, a putative antecedent of ASD (Brown et al., 2014; Koks et al., 2016; Meltzer & Van de Water, 2017), may occur more often in pregnancies that terminate prematurely. This might favor the appearance of a protective association of smoking with ASD in preterm cohorts as a result of collider stratification bias. Similarly, another form of collider stratification bias, resulting from the necessity of conditioning ASD assessments on live births, could induce a falsely protective association of smoking among preterm cohorts, given that tobacco smoke exposure in pregnancy is a risk factor for both premature birth and fetal loss (Liew et al., 2015; Pineles et al., 2014). The bias can occur if exposed children who are born preterm and do not survive were otherwise at a higher risk for ASD than those who did survive, potentially leading to a paradoxical protective association with smoking if it preferentially leads to fetal loss among fetuses susceptible to ASD (Goin et al., 2021), as described previously for air pollution (Leung et al., 2021; Raz et al., 2018). However, a key question with regard to ‘live‐birth bias’ is whether the interest lies in all conceptions or just among live births who reach the age when a diagnosis is possible, in which case, such bias is less likely or may be entirely moot.

Finally, the findings in preterm cohorts may also have been artifacts whereby: (1) the effect of smoking in such cohorts may not be comparable to effects in general population cohorts with a mix of pre‐ and (predominantly) full‐term births as demonstrated in a recent simulation study (Snowden & Basso, 2018); or (2) preterm birth could be on a causal pathway, in which case the effect measure in those cohorts will be a biased estimate of the total effect. Further analyses examining trimester‐specific maternal use of tobacco, mediation analysis of extreme prematurity, and maternal immune activation in preterm deliveries may shed further light on the unexpected results from preterm cohorts.

## CONCLUSION

In this meta‐analysis from U.S. cohorts participating in the ECHO program, maternal smoking anytime beginning 6 months prior to conception until delivery was consistently associated with higher dimensional measures of ASD traits (SRS scores). We also found a modest association with a diagnosis of ASD after excluding cohorts with likely biases from design features, or with small cell problems. The heterogeneity of results across diverse cohorts suggests the need for further investigations to examine the timing and amount of maternal smoking, adjust for additional potential confounders, and use objective measures of exposure. In the context of previously demonstrated risks from maternal smoking for other adverse child outcomes, our results suggest a potential additional benefit from smoking cessation programs and education—in medical care, nutrition counseling, government assistance programs and many more settings where messages can reach women of reproductive age, including difficult to reach groups. Effective outreach may help the development of social cognition and communication skills in the broader population of children.

## CONFLICT OF INTEREST

The authors have no conflicts of interest relevant to this article to disclose.

## Supporting information


**Supplemental Table 1** Sufficient adjustment sets for each cohort
**Supplemental Figure 1**. Directed Acyclic Graph (DAG)
**Supplemental Figure 2**. Association between maternal prenatal smoking and high SRS T‐scores (≥66) based on random effects model overall and by cohort type
**Supplemental Figure 3**. Statistical plots from the leave‐one‐out analysis for ASD
**Supplemental Figure 4**. Statistical plots from the leave‐one‐out analysis for the SRS.

## Data Availability

The datasets for this manuscript are not publicly available because, per the NIH‐approved ECHO (Environmental Influences on Child Health Outcomes) Data Sharing Policy, ECHO‐wide data have not yet been made available to the public for review/analysis. Requests to access the datasets should be directed to the ECHO Data Analysis Center, ECHO‐DAC@rti.org.
