# Supplementary material for: Maternal tobacco smoking and offspring autism spectrum disorder or traits in ECHO cohorts
Source: Autism Res. 2022 Feb 24;15(3):551–69. doi: 10.1002/aur.2665 (PMC9304219; doi:10.1002/aur.2665)
Supplement: Supplementary file 1 — Supplemental Table 1 Sufficient adjustment sets for each cohort Supplemental Figure 1. Directed Acyclic Graph (DAG) Supplemental Figure 2. Association between maternal prenatal smoking and high SRS T‐scores (≥66) based on random effects model overall and by cohort type Supplemental Figure 3. Statistical plots from the leave‐one‐out analysis for ASD Supplemental Figure 4. Statistical plots from the leave‐one‐out analysis for the SRS. [file AUR-15-551-s001.docx]

**Supplemental Table 1.** Sufficient adjustment sets for each cohort

| **Cohort #** | **Cohort Name** | **Sufficient adjustment sets** |
| --- | --- | --- |
| 1 | Healthy Start | Maternal diabetes, maternal high blood pressure, maternal age, maternal folic acid, maternal history of psychiatric disorders, maternal vitamin, neonatal complications, parity |
| 2 | Boricua Youth Study | Maternal education, maternal age |
| 3 | PETALS & KPRB | Time of initiation to health care access, maternal diabetes, maternal age, maternal folic acid, maternal history of psychiatric disorders, maternal vitamin, parity |
| 4 | [ReCHARGE](https://echoportal.org/MetadataCatalog/Cohorts/Details/41) | Time of initiation to health care access, maternal diabetes, maternal age, maternal folic acid, maternal history of psychiatric disorders, maternal vitamin, parity |
| 5 | CANDLE | Maternal alcohol use, maternal diabetes, maternal drug use, paternal history of psychiatric disorders, maternal high blood pressure, maternal age, maternal folic acid, maternal history of psychiatric disorders, maternal vitamin, neonatal complications, parity |
| 6 | ECHO-NOVI | Maternal education, maternal age, maternal history of psychiatric disorders |
| 7 | [Early Parenting of Children](https://echoportal.org/MetadataCatalog/Cohorts/Details/52) | Maternal education, maternal age, maternal history of psychiatric disorders |
| 8 | EARLI (Johns Hopkins University; University of California; Kaiser Permanente) | Maternal education, maternal age, maternal history of psychiatric disorders |
| 9 | Project Viva | Maternal education, maternal age, maternal history of psychiatric disorders |
| 10 | [Extremely Low Gestational Age Newborn](https://echoportal.org/MetadataCatalog/Cohorts/Details/73) | Maternal education, maternal age |
| 11 | [Inova Childhood Longitudinal Study](https://echoportal.org/MetadataCatalog/Cohorts/Details/87) | Maternal education, maternal age, maternal history of psychiatric disorders |
| 12 | [New Hampshire Birth Cohort Study](https://echoportal.org/MetadataCatalog/Cohorts/Details/45) | Maternal education, maternal age, maternal history of psychiatric disorders |
| 13 | ARCH | Maternal education, maternal age |

ARCH, Archive for Research on Child Health; CANDLE, Conditions Affecting Neurocognitive Development and Learning in Early Childhood; EARLI, Early Autism Risk Longitudinal Investigation; ECHO-NOVI, Environmental influences on Child Health Outcomes - Neonatal Neurobehavior and Outcomes in Very Preterm Infants; KPRB, Kaiser Permanente Research Bank; PETALS, Pregnancy Environment and Lifestyle Study; [ReCHARGE](https://echoportal.org/MetadataCatalog/Cohorts/Details/41), Revisiting CHildhood Autism Risks from Genes and the Environment Study.

**Supplemental Figure 1: Directed Acyclic Graph (DAG)**


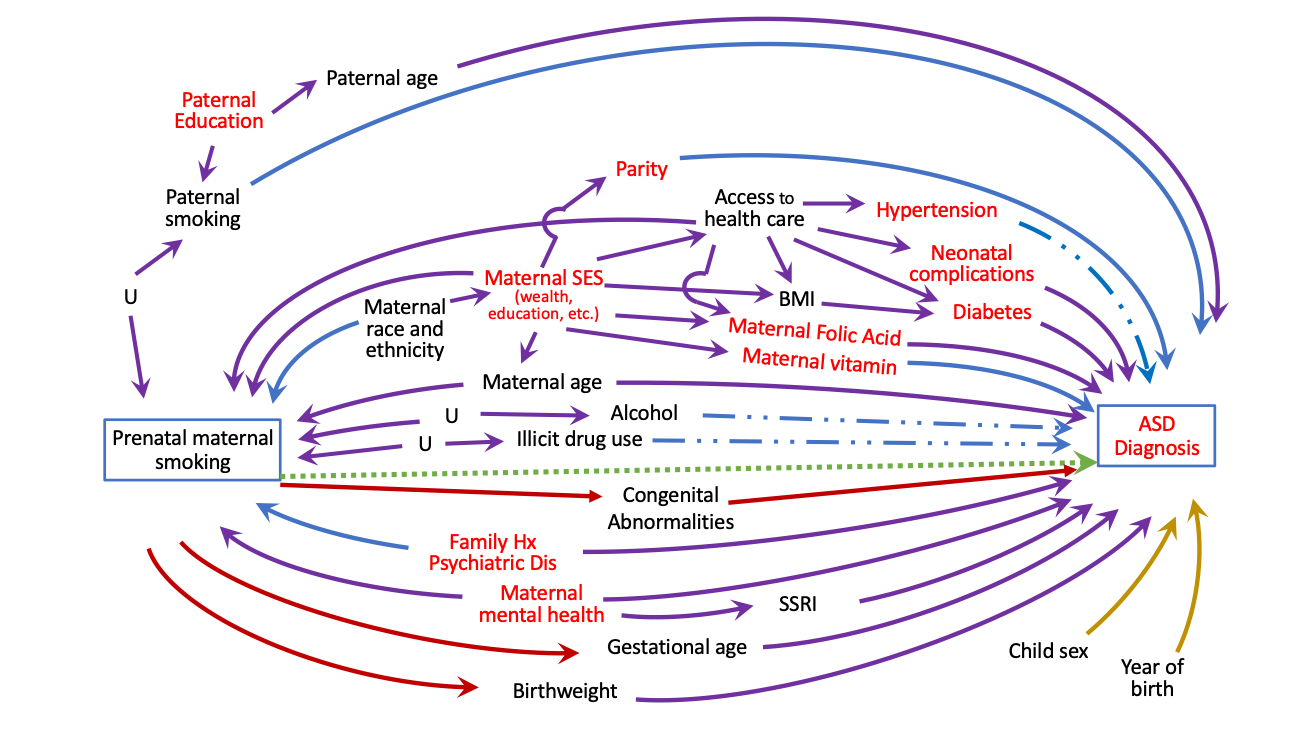


Legend to Supplemental Figure 1:

**Potential or Likely Confounders:**


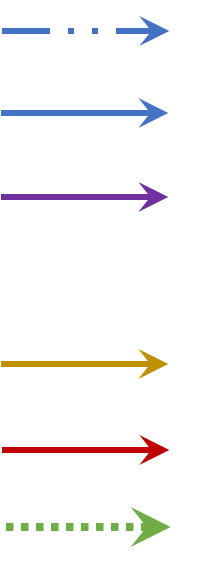
Weak evidence for association

Evidence for association

Substantial evidence for association

**Not Confounders:**

No association with exposure

Pathway to an intermediate variable

Study hypothesis

**References for DAG**

*Mental health and smoking*

Holtrop, J. S., Meghea, C., Raffo, J. E., Biery, L., Chartkoff, S. B., & Roman, L. (2010). Smoking among pregnant women with Medicaid insurance: are mental health factors related? *Maternal and Child Health Journal*, *14*(6), 971–977. https://doi.org/10.1007/s10995-009-0530-x

Lawrence, D., Mitrou, F., & Zubrick, S. R. (2009). Smoking and mental illness: results from population surveys in Australia and the United States. *BMC Public Health*, *9*, 285. https://doi.org/10.1186/1471-2458-9-285

Liu, D., Younger, E., Baker, S., Touch, S., Willmoth, T., & Hartos, J. L. (2019). Does current general mental health status relate to current smoking status in pregnant women? *Journal of Pregnancy*, *2019*, 7801465. https://doi.org/10.1155/2019/7801465

*Mental health and autism spectrum disorder (ASD)*

Cross-Disorder Group of the Psychiatric Genomics Consortium, Lee, S. H., Ripke, S., Neale, B. M., Faraone, S. V., Purcell, S. M., Perlis, R. H., Mowry, B. J., Thapar, A., Goddard, M. E., Witte, J. S., Absher, D., Agartz, I., Akil, H., Amin, F., Andreassen, O. A., Anjorin, A., Anney, R., Anttila, V., Arking, D. E., … International Inflammatory Bowel Disease Genetics Consortium (IIBDGC) (2013). Genetic relationship between five psychiatric disorders estimated from genome-wide SNPs. *Nature Genetics*, *45*(9), 984–994. <https://doi.org/10.1038/ng.2711>

Kodesh, A., Levine, S. Z., Khachadourian, V., Rahman, R., Schlessinger, A., O'Reilly, P. F., Grove, J., Schendel, D., Buxbaum, J. D., Croen, L., Reichenberg, A., Sandin, S., & Janecka, M. (2021). Maternal health around pregnancy and autism risk: a diagnosis-wide, population-based study. *Psychological Medicine*, 1–9. Advance online publication. https://doi.org/10.1017/S0033291721001021

Liang, C. S., Bai, Y. M., Hsu, J. W., Huang, K. L., Ko, N. Y., Yeh, T. C., Chu, H. T., Tsai, S. J., Chen, T. J., & Chen, M. H. (2021). Associations of parental mental disorders and age with childhood mental disorders: a population-based cohort study with four million offspring. *European Child & Adolescent Psychiatry*, 10.1007/s00787-021-01914-3. Advance online publication. https://doi.org/10.1007/s00787-021-01914-3

*Alcohol & ASD*

Gallagher, C., McCarthy, F. P., Ryan, R. M., & Khashan, A. S. (2018). Maternal alcohol consumption during pregnancy and the risk of autism spectrum disorders in offspring: a retrospective analysis of the millennium cohort study. *Journal of Autism and Developmental Disorders*, *48*(11), 3773–3782. https://doi.org/10.1007/s10803-018-3626-6

Singer, A. B., Aylsworth, A. S., Cordero, C., Croen, L. A., DiGuiseppi, C., Fallin, M. D., Herring, A. H., Hooper, S. R., Pretzel, R. E., Schieve, L. A., Windham, G. C., & Daniels, J. L. (2017). Prenatal alcohol exposure in relation to autism spectrum disorder: findings from the Study to Explore Early Development (SEED). *Paediatric and Perinatal Epidemiology*, *31*(6), 573–582. https://doi.org/10.1111/ppe.12404

*Maternal diabetes and ASD*

Hertz-Picciotto, I., Schmidt, R. J., & Krakowiak, P. (2018). Understanding environmental contributions to autism: Causal concepts and the state of science. *Autism Research : Official Journal of the International Society for Autism Research*, *11*(4), 554–586. https://doi.org/10.1002/aur.1938

Xiang, A. H., Wang, X., Martinez, M. P., Walthall, J. C., Curry, E. S., Page, K., Buchanan, T. A., Coleman, K. J., & Getahun, D. (2015). Association of maternal diabetes with autism in offspring. *JAMA*, *313*(14), 1425–1434. https://doi.org/10.1001/jama.2015.2707

Xu, G., Jing, J., Bowers, K., Liu, B., & Bao, W. (2014). Maternal diabetes and the risk of autism spectrum disorders in the offspring: a systematic review and meta-analysis*. Journal of Autism and Developmental Disorders*, *44*(4), 766–775. https://doi.org/10.1007/s10803-013-1928-2

*Maternal or paternal age and ASD*

Janecka, M., Mill, J., Basson, M. A., Goriely, A., Spiers, H., Reichenberg, A., Schalkwyk, L., & Fernandes, C. (2017). Advanced paternal age effects in neurodevelopmental disorders-review of potential underlying mechanisms. *Translational Psychiatry*, *7*(1), e1019. https://doi.org/10.1038/tp.2016.294

Sandin, S., Hultman, C. M., Kolevzon, A., Gross, R., MacCabe, J. H., & Reichenberg, A. (2012). Advancing maternal age is associated with increasing risk for autism: a review and meta-analysis. *Journal of the American Academy of Child and Adolescent Psychiatry*, *51*(5), 477–486.e1. https://doi.org/10.1016/j.jaac.2012.02.018

Shelton, J. F., Tancredi, D. J., & Hertz-Picciotto, I. (2010). Independent and dependent contributions of advanced maternal and paternal ages to autism risk. *Autism Research: Official Journal of the International Society for Autism Research*, *3*(1), 30–39. https://doi.org/10.1002/aur.116

*Maternal age and smoking*

Mohlman, M. K., & Levy, D. T. (2016). Disparities in maternal child and health outcomes attributable to prenatal tobacco use. *Maternal and Child Health Journal*, *20*(3), 701–709. https://doi.org/10.1007/s10995-015-1870-3

*Maternal socioeconomic status (SES) and prenatal smoking*

Houston-Ludlam, A. N., Bucholz, K. K., Grant, J. D., Waldron, M., Madden, P., & Heath, A. C. (2019). The interaction of sociodemographic risk factors and measures of nicotine dependence in predicting maternal smoking during pregnancy. *Drug and Alcohol Dependence*, *198*, 168–175. https://doi.org/10.1016/j.drugalcdep.2019.02.014

*Race/ethnicity and SES*

U.S. Census 2020: Table 3. Detailed years of school completed by people 25 years and over by sex, age groups, race and Hispanic origin: 2020. <https://www.census.gov/data/tables/2020/demo/educational-attainment/cps-detailed-tables.html>

[Wight](https://www.nccp.org/author/vanessa-r-wight/), V. R., [Chau](https://www.nccp.org/author/michelle-chau/), M., [& Aratani](https://www.nccp.org/author/yumiko-aratani/), Y. (2011). Who are America’s poor children? The official story. *National Center for Children in Poverty*. [http://www.nccp.org/publication/who-are-americas-poor-children-the-official-story/](https://nam12.safelinks.protection.outlook.com/?url=http%3A%2F%2Fwww.nccp.org%2Fpublication%2Fwho-are-americas-poor-children-the-official-story%2F&data=04%7C01%7Ciher%40ucdavis.edu%7C7297d9e43bf249554f2d08d9b38d4880%7Ca8046f6466c04f009046c8daf92ff62b%7C0%7C0%7C637738241708781011%7CUnknown%7CTWFpbGZsb3d8eyJWIjoiMC4wLjAwMDAiLCJQIjoiV2luMzIiLCJBTiI6Ik1haWwiLCJXVCI6Mn0%3D%7C3000&sdata=HJVHMPrako%2FCPgNA7bZ%2B6J8DoHLUbJeP7eV%2Babfe%2BqQ%3D&reserved=0)

Williams, D. R. (1996). Race/ethnicity and socioeconomic status: measurement and methodological issues. *International Journal of Health Services: Planning, Administration, Evaluation*, *26*(3), 483–505. https://doi.org/10.2190/U9QT-7B7Y-HQ15-JT14

Williams, D. R., Mohammed, S. A., Leavell, J., & Collins, C. (2010). Race, socioeconomic status, and health: complexities, ongoing challenges, and research opportunities. *Annals of the New York Academy of Sciences,* *1186*, 69–101. https://doi.org/10.1111/j.1749-6632.2009.05339.x

*SES and folic acid intake during pregnancy*

Branum, A. M., Bailey, R., & Singer, B. J. (2013). Dietary supplement use and folate status during pregnancy in the United States. *The Journal of Nutrition*, *143*(4), 486–492. https://doi.org/10.3945/jn.112.169987

Sullivan, K. M., Ford, E. S., Azrak, M. F., & Mokdad, A. H. (2009). Multivitamin use in pregnant and nonpregnant women: results from the Behavioral Risk Factor Surveillance System. *Public Health Reports (Washington, D.C.: 1974)*, *124*(3), 384–390. https://doi.org/10.1177/003335490912400307

*Folic acid and ASD*

Hoxha, B., Hoxha, M., Domi, E., Gervasoni, J., Persichilli, S., Malaj, V., & Zappacosta, B. (2021). Folic acid and autism: a systematic review of the current state of knowledge. *Cells*, *10*(8), 1976. https://doi.org/10.3390/cells10081976

Levine, S. Z., Kodesh, A., Viktorin, A., Smith, L., Uher, R., Reichenberg, A., & Sandin, S. (2018). Association of maternal use of folic acid and multivitamin supplements in the periods before and during pregnancy with the risk of autism spectrum disorder in offspring. *JAMA Psychiatry*, *75*(2), 176–184. https://doi.org/10.1001/jamapsychiatry.2017.4050

Surén, P., Roth, C., Bresnahan, M., Haugen, M., Hornig, M., Hirtz, D., Lie, K. K., Lipkin, W. I., Magnus, P., Reichborn-Kjennerud, T., Schjølberg, S., Davey Smith, G., Øyen, A. S., Susser, E., & Stoltenberg, C. (2013). Association between maternal use of folic acid supplements and risk of autism spectrum disorders in children. *JAMA*, *309*(6), 570–577. https://doi.org/10.1001/jama.2012.155925

*Maternal SES and parity*

Hamilton B. E. (2021). 2021 Total fertility rates, by maternal educational attainment and race and Hispanic origin: United States, 2019. *National Vital Statistics Reports: from the Centers for Disease Control and Prevention, National Center for Health Statistics, National Vital Statistics System*, *70*(5), 1–9.

Musick, K., England, P., Edgington, S., & Kangas, N. (2009). Education differences in intended and unintended fertility. *Social Forces; A Scientific Medium of Social Study and Interpretation*, *88*(2), 543–572. https://doi.org/10.1353/sof.0.0278

*Access to health care in relation to smoking, hypertension, diabetes, neonatal complications*

Hackshaw, A., Rodeck, C., & Boniface, S. (2011). Maternal smoking in pregnancy and birth defects: a systematic review based on 173 687 malformed cases and 11.7 million controls. *Human Reproduction Update*, *17*(5), 589–604. https://doi.org/10.1093/humupd/dmr022

Leite, M., Albieri, V., Kjaer, S. K., & Jensen, A. (2014). Maternal smoking in pregnancy and risk for congenital malformations: results of a Danish register-based cohort study. *Acta Obstetricia et Gynecologica Scandinavica*, *93*(8), 825–834. https://doi.org/10.1111/aogs.1243

Marufu, T. C., Ahankari, A., Coleman, T., & Lewis, S. (2015). Maternal smoking and the risk of still birth: systematic review and meta-analysis. *BMC Public Health*, *15*, 239. https://doi.org/10.1186/s12889-015-1552-5

Moore, T. R., Origel, W., Key, T. C., & Resnik, R. (1986). The perinatal and economic impact of prenatal care in a low-socioeconomic population. *American Journal of Obstetrics and Gynecology*, *154*(1), 29–33. <https://doi.org/10.1016/0002-9378(86)90387-x>

Musonge-Effoe, J.E., Alema-Mensah. E., Effoe, V.S., Akinnawo, F., Caplan, L. (2020). The association between health care coverage and prevalence of cardiovascular diseases and diabetes over a 10-year period. *Prev Med*icine, 132:105983. doi: 10.1016/j.ypmed.2020.105983. Epub 2020 Jan 16. PMID: 31954838.

Nussey, L., Hunter, A., Krueger, S., Malhi, R., Giglia, L., Seigel, S., Simpson, S., Wasser, R., Patel, T., Small, D., & Darling, E. K. (2020). Sociodemographic characteristics and clinical outcomes of people receiving inadequate prenatal care: a retrospective cohort study. *Journal of Obstetrics and Gynaecology Canada: JOGC = Journal d'Obstetrique et Gynecologie du Canada: JOGC*, *42*(5), 591–600. https://doi.org/10.1016/j.jogc.2019.08.005

**Supplemental Figure 2. Association between maternal prenatal smoking and high SRS T-scores (≥66) based on random effects model overall and by cohort type**


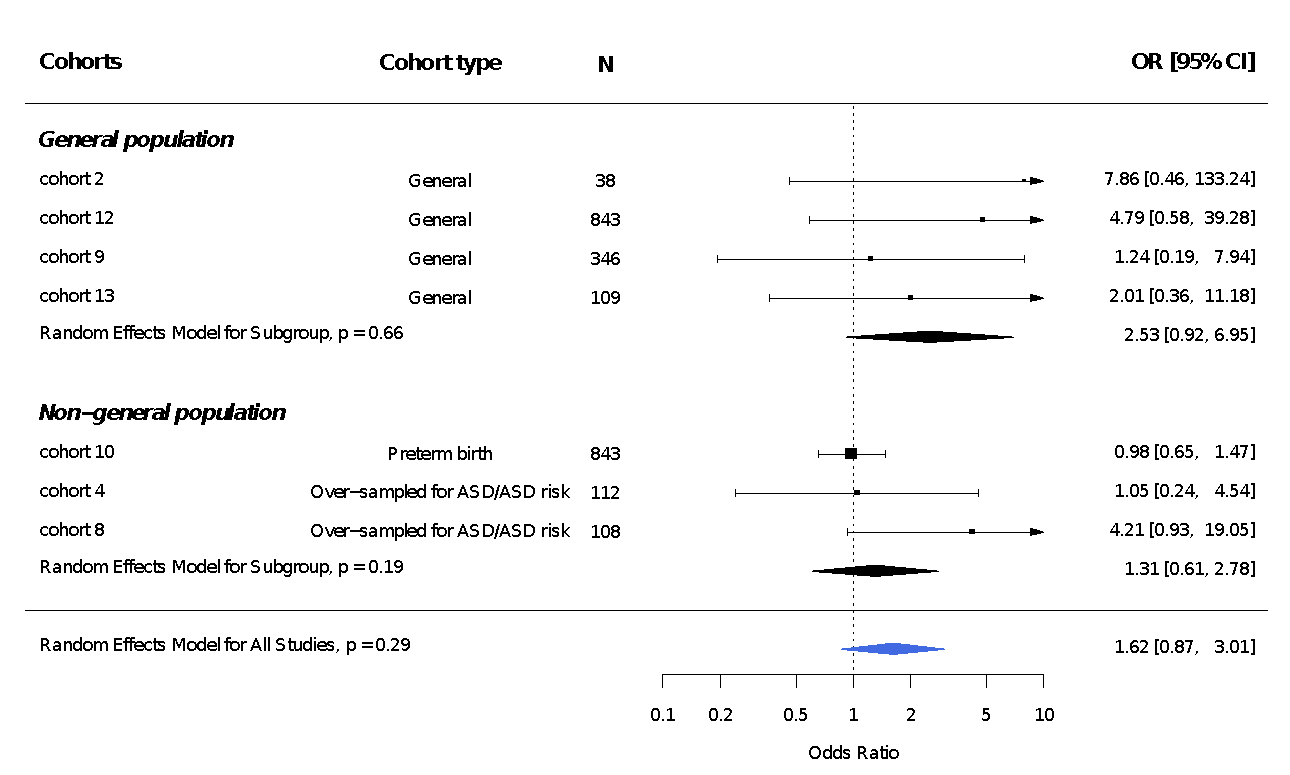


Participants with non-missing values in cohort-specific sufficient adjustment sets were included in the pooled analysis. The sample sizes listed represent the total sample included in the meta-analysis; this may differ from those listed in Table 2 based on missing data. ASD, autism spectrum disorder; SRS, Social Responsiveness Scale.

**Supplemental Figure 3. Statistical plots from the leave-one-out analysis for ASD**

ASD, autism spectrum disorder.

**Supplemental Figure 4. Statistical plots from the leave-one-out analysis for the SRS**


SRS, Social Responsiveness Scale.
